# Effects of Physical Activity, Metabolic Syndrome, and Social Status on ECG Parameters in Children: A Prospective Cohort Study

**DOI:** 10.3390/jcdd12100385

**Published:** 2025-09-29

**Authors:** Árpád Kézdi, Viktor József Horváth, Regina Hangács, Ádám Gyula Tabák, Dominic Joseph Fogarasi, Dániel Vadon, György Grósz, Ferenc Fekete, Anikó Nagy

**Affiliations:** 1 Department of Internal Medicine and Oncology, Semmelweis University, H-1097 Budapest, Hungary; kezdi.arpad@semmelweis.hu (Á.K.); tabak.adam@semmelweis.hu (Á.G.T.); fog.domos@gmail.com (D.J.F.); vadondaniel00@gmail.com (D.V.); 2 Heim Pál National Pediatric Institute, Üllői út 86, H-1089 Budapest, Hungary; hangacs.regina@gmail.com (R.H.); efekete@heimpalkorhaz.hu (F.F.); foig@heimpalkorhaz.hu (A.N.); 3 Institute of Preventive Medicine and Public Health, Semmelweis University, H-1097 Budapest, Hungary; 4 MSB-MET Ltd., H-8230 Balatonfüred, Hungary; ggrosz@msbmet.com

**Keywords:** children, resting ECG, metabolic syndromre, sport activity, social status

## Abstract

(1) Background: Physical activity, altered metabolic parameters, and socio-economic status may affect electrocardiographic (ECG) parameters in children. However, a direct comparison of their effects on resting ECG has not yet been performed. (2) Methods: A total of 139 participants (60 male), aged 10–17 years, were recruited. Resting 1-minute ECG recordings and clinical and laboratory investigations were obtained, while socio-economic status and physical activity were assessed using a questionnaire. Associations between these factors and ECG parameters were analyzed using analysis of covariance (ANCOVA). (3) Results: Age, sex, metabolic syndrome, and physical activity significantly influenced the average RR interval (η^2^ = 0.292, 0.070, 0.078, and 0.070, respectively). Similar effects were observed on the T_end–P interval. The PR, QRS, QTc, and T_peak–T_end intervals were moderately influenced by age (η^2^ = 0.084, 0.056, 0.072, and 0.049, respectively). QTc was additionally affected by sex (η^2^ = 0.060). None of the modifiable factors had any effect on depolarization or repolarization parameters. Socio-economic status had no significant effect on resting ECG. (4) Conclusions: Physical activity exerts similar effects on resting ECG in both sexes, while metabolic syndrome is an independent determinant of several ECG parameters. Further studies are warranted to clarify the clinical relevance of these findings.

## 1. Introduction

Electrocardiography (ECG) is a widely used non-invasive method for assessing cardiac electrical activity. It provides essential information for diagnosing various cardiac disorders (e.g., long QT syndrome, Brugada syndrome, Wolff–Parkinson–White syndrome) while also aiding in the management of systemic conditions.

Resting ECG values are influenced by several factors. First, metabolic syndrome (MS) and its components can affect ECG parameters as early as childhood [1,2]. Second, physical activity (PA) exerts beneficial effects on the cardiovascular system in children, which are also reflected in certain resting ECG parameters [3,4,5]. Third, children who experience adverse socio-economic circumstances—independently of their conditions in adulthood—are generally at higher risk of developing cardiovascular diseases [6], although a direct link between socio-economic status (SES) and childhood ECG has not been clearly established. In addition, age influences multiple ECG parameters in both children and adults [7].

These considerations highlight the importance of examining the combined effects of MS, PA, and SES on resting ECG. While the individual impacts of these factors have been reported in numerous studies, their simultaneous influence on and potential interactions with resting ECG parameters in children remain largely unexplored. Therefore, this study aimed to evaluate the combined impact of MS, PA, and SES on resting ECG parameters in children while accounting for age as a physiological determinant of ECG values.

## 2. Methods

This prospective cohort study was conducted on children at Heim Pál Hospital, Budapest, Hungary, between January 2023 and January 2025. This study was conducted in accordance with the Declaration of Helsinki. Ethical approval was obtained from the Medical Ethics Research Council of Hungary with the number OGYÉI/56533-3/2023. All patients’ legal representatives (in all cases, one of the parents) signed an informed consent form and agreed to the scientific usage of the collected data.

### 2.1. Study Participants

This study included randomly selected pediatric patients from the HOGYI questionnaire (protocol number: HOGYI/TKP 2023; project ID at AdWare Research: 1095CCL/2022). The HOGYI questionnaire is part of a nationwide survey designed to assess children’s mental and physical health. For the present study, only those items relevant to Section 3.3 of the public education questionnaire were analyzed.

Data from 22,808 children were available in the database. Using a random number generator, 1000 patients were invited to participate, of whom 177 responded and agreed to take part in this study. The exclusion criteria were as follows: inability to cooperate, as judged by the examining physician (e.g., due to mental condition); fever within 10 days before the examination; any surgical intervention within the previous 3 months; body weight change exceeding 15% in either direction during the month before the examination; a known congenital heart malformation (except for a persistent patent foramen ovale considered hemodynamically insignificant in a prior cardiology assessment, surgically corrected patent ductus arteriosus, or coarctation of the aorta); hemodynamically significant acquired or structural heart disease; or paroxysmal supraventricular tachycardia or atrial fibrillation/flutter in their medical history. Additional exclusion criteria included uncontrolled thyroid disease, malnutrition, anemia (hemoglobin < 110 g/L for girls; <120 g/L for boys), or a history of unexplained loss of consciousness. None of the participants were taking cardiovascular medication. Informed consent was obtained in person from each participant’s legal representative (in all cases, one of the parents). Demographic data (age and sex), physical activity level, socio-economic status, and information on current and past chronic illnesses were collected using the questionnaire and transferred to a standardized data entry form.

### 2.2. Baseline Assessment

A detailed physical examination and laboratory testing were performed by an accredited laboratory and a medical professional after a minimum of 12 h of fasting. The following parameters were measured: fasting blood glucose (mmol/L), HbA1c (% and mmol/mol), total cholesterol (mmol/L), HDL cholesterol (mmol/L), and triglycerides (mmol/L). LDL cholesterol was calculated using the Friedewald formula (LDL cholesterol = total cholesterol-HDL cholesterol-[triglycerides/5]). Height (cm, to the nearest 0.1 cm) and weight (kg, to the nearest 0.1 kg) were measured using standard methods but were not used in this analysis (data available upon request). Following World Health Organization recommendations [8], waist circumference (WC) was measured midway between the lowest rib and the superior border of the iliac crest at the end of normal expiration using a flexible, nonelastic anthropometric tape (to the nearest 0.1 cm). Blood pressure was measured in the sitting position using an Omron M2 device, after at least 5 min of rest, on both arms. The measurement was repeated on the arm with the higher value, and the mean of the two readings was reported. If the value exceeded the 95th percentile for age and sex, the patient was excluded and referred to a specialist.

Parameters related to metabolic syndrome (MS) were combined into a single variable used to classify participants. Although formal diagnosis of MS in children is not recommended before the age of 10, risk factors are often present earlier and should be addressed before diagnosis [9]. In contrast with the adult definition, only two of the following criteria are sufficient to diagnose MS in children [9]: WC ≥ the 90th percentile for age and sex; triglycerides ≥ 1.69 mmol/L (150 mg/dL); HDL cholesterol < 1.03 mmol/L (40 mg/dL); fasting blood glucose > 5.6 mmol/L (100 mg/dL); or known type 2 diabetes mellitus.

Physical activity (PA) was assessed with the following question: “Apart from school physical education, how much does your child exercise every day?” Responses were scored as follows: “not at all” or “less than 30 min” scored 0 points (no regular exercise); “30–60 min” or “60–90 min” scored 1 point (occasional exercise); and “more than 90 min” scored 2 points (regular exercise).

Socio-economic status (SES) was assessed using a weighted questionnaire. Scores were assigned as follows. Parental education: both parents with primary education, or one with primary and the other with secondary education = 0 points; both parents with higher education (BSc, MSc, PhD, or equivalent) = 2 points; and all other cases = 1 point. Car ownership: 0 cars = 0 points; 1 car = 1 point; and >1 car = 2 points. Vacation frequency: none = 0 points; once per year = 1 point; and more than once = 2 points. Parents’ perception of social status: “below average” = 0 points; “average” = 1 point; and “above average” = 2 points.

### 2.3. ECG Measurements

ECGs were recorded by trained technicians according to a standardized 12-lead acquisition protocol. Participants rested in the supine position for at least 5 min before the 1-min recording began. ECGs were obtained using the CPNSS/DB integrated cardiovascular and sensory nerve neuropathy measurement system (MSB-MET Ltd., Balatonfüred, Hungary) at a sampling frequency of 500 Hz. Raw ECG data were exported electronically without filtering. Standard calibration was applied (10 mm = 1 mV; 25 mm/s).

All ECGs were evaluated electronically by a medical professional. The following parameters were assessed: RR interval, PR interval, QRS duration, QTc (Bazett’s correction), T_peak–T_end (TTe), and T_end–P (TP) intervals (all in milliseconds). If the value of a single cardiac cycle significantly deviated from the average (> 2 SD from the mean), the cycle was checked manually in the original recording. ECGs with technical issues, such as poor baseline quality or missing lead information, were excluded. If a resting ECG revealed abnormalities (> 150/min tachycardia, <40/min bradycardia, extrasystoles during recording, or conduction abnormalities), the patient was excluded from further analysis and referred for cardiology evaluation.

**Figure 1:**
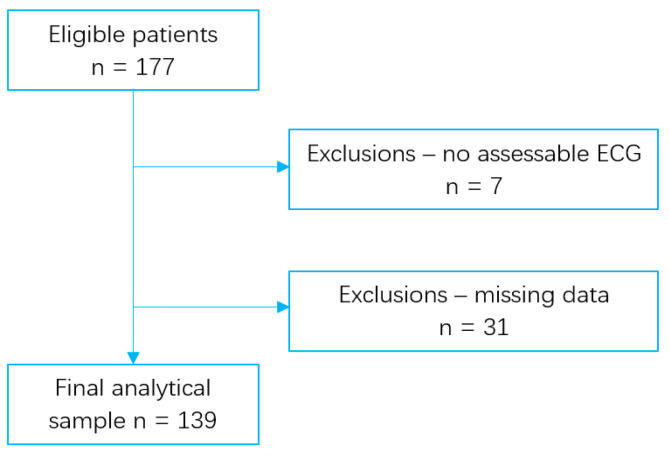
Flowchart of participants’ selection.

Figure 1 shows that a total of 177 patients were eligible for inclusion. Of these, 7 had non-assessable ECGs due to technical issues, and 31 were excluded because of missing data. The remaining 139 patients were included in the final analysis.

### 2.4. Statistical Methods

Statistical analyses were performed using SPSS Statistics, version 29.0 (IBM Corp., Armonk, NY, USA). Analysis of covariance (ANCOVA) was conducted with the RR interval, PR interval, QRS duration, QTc interval, TTe, and TP intervals as dependent variables. Sex, metabolic syndrome (MS), and socio-economic status (SES) were entered as fixed factors, while age was included as a covariate. The model was applied to evaluate the main effects of each factor as well as pairwise interactions. Assumptions of normality and homogeneity of variance were tested. Statistical significance was defined as *p* < 0.05.

## 3. Results

Of the 139 participants included in the analysis, 60 were male. Age, diastolic blood pressure, serum triglycerides, HDL cholesterol, and fasting glucose levels did not differ significantly between sexes. However, male participants had a higher waist circumference and slightly higher systolic blood pressure, although both values remained within the normal range and were of limited clinical relevance. In contrast, no differences were observed between sexes in physical activity (PA) or socio-economic status (SES). Baseline characteristics are presented in Table 1.

**Figure 2:**
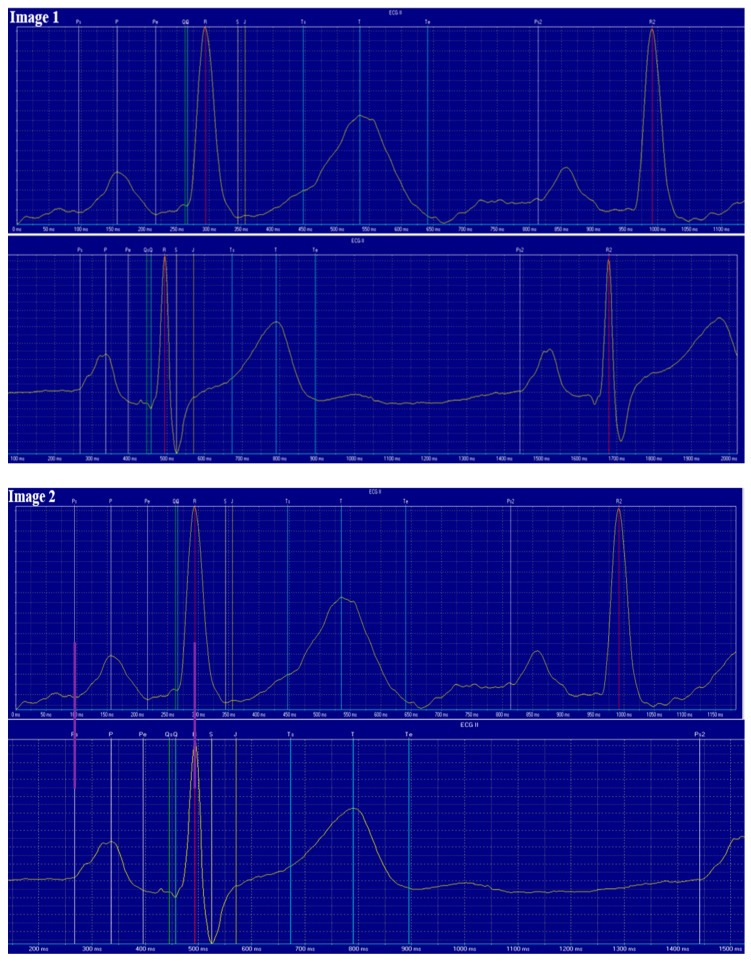
Representative images of two participants. See detalied explanation in the text.

ANCOVA was applied to evaluate the effects of different factors on resting ECG parameters. For easier interpretation, representative ECG tracings at different heart rates are shown in Figure 2. Heart-rate-related parameters, specifically the RR and TP intervals, were strongly influenced by all examined factors, except SES (Table 2). The strongest association was observed between age and the RR or TP interval. Sex and metabolic syndrome (MS) had similar effects on these intervals, while estimated physical activity (PA) showed the weakest, though still statistically significant, effect.

ANCOVA was also performed for conduction-, depolarization-, and repolarization-related parameters (PR, QRS, QTc, and TTe). These were associated only with age, except for QTc, which was additionally influenced by sex, albeit weakly. Neither MS nor PA was associated with these ECG parameters, and SES was, again, not a significant explanatory factor. No two-way interactions between factors reached statistical significance. Detailed results are presented in Table 2.

In Image 1, the upper panel shows a 17-year-old participant with a heart rate of 86/min and no regular physical activity, while the lower panel depicts a 16-year-old participant with a heart rate of 52/min who engaged in regular physical activity. The scaling is identical, and the recordings are aligned at the onset of the P-waves. The most notable difference is in the T_end–P interval, which is longer at the lower heart rate (lower panel).

Image 2 displays recordings from the same two participants after rescaling the lower ECG (HR: 52/min) so that the interval between the onset of the P-wave and the peak of the R-wave is equal. A purple line was added to aid visual interpretation. Comparison shows that the PR, QRS, QT, and TTe intervals are of a similar length, while the T_end-P and RR intervals are prolonged in the participant with the lower heart rate. The second QRS complex in the lower panel is not visible.

Next, the weighted impact of the variance of the investigated effects was calculated, which gives an estimation of what percentage of the variance can be explained by a certain parameter. By applying this approach to the RR interval, it was determined that age, gender, MS, PA, and SES altogether explained approximately R^2^ = 0.337 (34%) of the experienced variance. The same analysis for the TP value obtained R^2^ = 0.307 (31%). However, for the conduction, depolarization, and repolarization parameters, the weights of the investigated factors in the experienced variance were low or negligible (R^2^ values: PR—0.091 (9%), QRS—0.042 (4%), QTc—0.114 (11%), and TTe—0.090 (9%)).

After the main effects of the different conditions on resting ECG parameters were evaluated, we wanted to determine the numeric impact of these significant factors on the investigated parameters. Since age was used as a covariate, we filtered out its effect on ECG parameters by fixing it at its mean value (mean age: 12.98 years). After this, the average RR interval was 853 msec in male participants and 788 msec in female participants. This shows that an average difference of 65 msec could be explained by sex, with females exhibiting a higher resting heart rate. The presence of MS explained a similar effect, with a 74 msec difference (with an average RR of 858 msec in healthy participants and 784 msec in patients with MS). Independent of the duration of regular PA, its effect on heart rate-associated parameters was significant, although the duration of PA performed affected the values. Numerically, the difference between individuals performing regular 30–90 min of daily PA and those who did not perform regular PA was 49 msec (with average RR intervals of 770 msec vs. 819 msec for <30 min of PA vs. 30–90 min/day of physical activity, respectively). The difference between those performing >90 min/day of physical activity and those without regular PA was 104 msec (with average RR intervals of 770 msec vs. 874 msec). The data are summarized in Table 3.

The TP values were significantly influenced by the same factors. The numerical results were as follows: the average TP was 336 ms in males vs. 272 ms in females (Δ = 64 ms); 333 ms in participants without MS vs. 275 ms in those with MS (Δ = 58 ms); and 267, 306, and 340 ms in participants with <30, 30–90, and >90 min of daily PA, respectively (Δ = 39 and 73 ms compared with <30 min/day). These findings are summarized in Table 3.

Among the other ECG parameters, PR, QRS, QTc, and TTe intervals were all significantly affected by age. QTc was also influenced by sex. After adjusting for age at its mean value, sex accounted for an approximately 10 ms difference, with females having slightly higher values (average QTc: 413 ms in males vs. 423 ms in females). This difference was not considered clinically relevant. The detailed results are shown in Table 2.

## 4. Discussion

Resting ECG is a widely used non-invasive tool for evaluating cardiac electrical function in both adults and children. However, the combined effects of clinically important factors such as physical activity (PA), metabolic syndrome (MS), and socio-economic status (SES) on resting ECG values in children have not been systematically examined. Modern medicine increasingly emphasizes prevention rather than the treatment of manifest disease, making it crucial to identify long-term conditions that may influence easily measurable cardiovascular markers.

In our study, age strongly influenced RR, PR, QRS, QTc (Bazett’s correction), TTe, and TP intervals. Sex also exerted significant but differential effects on RR, QTc, and TP intervals. MS-related factors and PA primarily affected heart-rate-related markers (RR and TP), whereas SES showed no significant impact on resting ECG. QTc and TTe intervals, widely regarded as predictors of sudden cardiac death [10,11] and atrial fibrillation in adults [12], appeared relatively unaffected by these factors during childhood. Furthermore, no interactions between the investigated conditions were identified.

Resting ECG has been proposed as a screening tool in asymptomatic populations because it is reproducible, accessible, and non-invasive. However, cost-effectiveness is questionable, and routine use may lead to unnecessary testing and downstream interventions [13]. Due to insufficient evidence that screening improves outcomes, both the US Preventive Services Task Force [14] and the European Society of Cardiology [15] advise against ECG screening in asymptomatic adults at low cardiovascular risk. In contrast, competitive athletes benefit from screening with resting ECGs, given that (1) the baseline incidence of sudden cardiac death is higher among competitive athletes compared with the general population and that (2) a routine ECG screening program was associated with an 89% reduction in mortality [16]. This recommendation is endorsed by the recent Italian COCIS guidelines on competitive sport eligibility [17].

Childhood chronic diseases such as obesity are known to adversely affect cardiovascular structure and function. Pediatric obesity is associated with long-term consequences and an increased risk of major cardiovascular risk factors later in life [18]. Previous studies have shown that MS is associated with a wide range of ECG alterations, including PR prolongation [1], QT/QTc changes [19,20,21], TTe prolongation [22] in adults, and increased heart rate in children [2]. Exercise also induces typical ECG changes in children [23], and differences in ECG parameters have been observed even among athletes of different pediatric age groups [24]. These observations support the rationale for performing resting ECGs for both screening and follow-up in pediatric populations.

Among MS components, obesity is one of the most extensively studied risk factors influencing ECG [2,4]. It is easily assessed in clinical practice and has well-documented effects on ECG, particularly in adults. Subtle ECG changes observed in obese children may progress into pathological alterations in adulthood [3]. Two meta-analyses demonstrated that an elevated childhood BMI is associated with an increased risk of coronary heart disease in later life, including stable angina and myocardial infarction [25,26]. Prolonged QT intervals, increased QT dispersion, and longer JT intervals have been reported in obese children with MS [27]. Another systematic review concluded that obese children exhibit only marginal QTc prolongation compared with non-obese peers, and even this prolongation generally remains within the normal range [28]. Taken together, the effects of pediatric obesity on ECG parameters are diverse.

The definition of obesity in childhood is heterogeneous, and it is difficult to isolate single components of MS to analyze their impact on ECG. These considerations emphasize the importance of evaluating metabolic factors early in life, as ECG alterations observed in childhood may represent early markers of future cardiovascular disease [18,25,26,29]. Although beyond the scope of the present cross-sectional study, it is plausible that related conditions, such as regular PA, MS, and sedentary behavior, leave characteristic imprints on childhood ECG that may predict future cardiovascular risk. Continuing this line of reasoning, the evaluation of PA deserves special attention, since regular PA is clearly linked to beneficial electrophysiological, structural, and functional cardiac adaptations in children [30]. While further studies are needed to clarify which specific factors (e.g., growth, race, and sex) drive ECG changes in pediatric athletes, the existing data show that young athletes have longer PR intervals and higher frequencies of sinus bradycardia, first-degree atrioventricular block, and incomplete right bundle branch block [31]. They also more often meet voltage criteria for left-ventricular hypertrophy and display T-wave inversions, particularly in the inferolateral leads. These findings are mediated by age, race, and sex. Although our results cannot be directly compared with these observations due to differences in PA definitions, the simplicity and reproducibility of our questionnaire-based PA assessment represent a practical strength.

Another area of interest is the relationship between cardiometabolic risk, PA, and heart rate variability (HRV) [30,32]. Multiple approaches have been used to evaluate this association, and reviews focusing on pediatric populations confirm that reduced HRV reflects impaired autonomic regulation, which is linked to higher cardiometabolic risk and mortality [33]. Meta-analyses further indicate that greater PA in children and adolescents is associated with higher HRV [34]. Direct comparison with our findings is not possible, since the 1-minute resting ECG recordings used here were insufficient to estimate HRV, allowing only calculation of heart rate standard deviation. Nevertheless, our results demonstrate that even shorter durations of PA exert measurable effects on heart rate, underscoring the value of promoting regular PA in children.

With respect to SES, our study found no association with resting ECG parameters. Recent work has emphasized the impact of early-life developmental conditions on adult cardiovascular outcomes, particularly in ethnic minority populations [35,36,37]. The key biological mechanism underlying the link between low SES and adverse health outcomes is chronic stress-related activation of the autonomic nervous system [36]. Consequently, a higher resting heart rate might be expected in children with a lower SES. However, we observed no such differences, possibly due to the limited sensitivity of our SES questionnaire. More nuanced measures, addressing optimism, purpose, or self-esteem, have been associated with better long-term outcomes [38], but such questions are impractical in routine clinical settings. SES may still exert indirect effects on ECG through its influence on PA, body weight, and diet, but these effects may not yet manifest in early childhood.

Several limitations of our study should be noted. Although correction of QT for heart rate is recommended, there is debate regarding the optimal method [39]. Similar to much of the existing literature, we applied Bazett’s formula, the most widely used approach. As a cross-sectional study, we cannot assess predictive value, and the true clinical implications of our findings remain to be determined. The short duration of the ECG recordings (1 min) precluded HRV analysis. Age-related developmental effects on ECG parameters could not be fully excluded. Finally, although unlikely, selection bias may have occurred if healthier, more motivated families were more likely to participate.

Despite these limitations, our study has several strengths. The 1-minute-resting ECG protocol proved clinically feasible, easy to implement, and highly reproducible. The recording system enabled precise measurements by saving each beat separately and allowing manual adjustments when necessary. No extrasystoles or overt conduction or repolarization abnormalities were detected, yielding a relatively homogeneous study population. Furthermore, the availability of detailed clinical data minimized exclusions and reduced the likelihood of bias, thereby strengthening the robustness of our results.

In summary, our study provides new insights into the value of resting ECG parameters in children across different ages. QTc and PR intervals appeared relatively stable, regardless of the presence of MS, while SES, as assessed by our questionnaire, showed no impact. Future studies should clarify whether HRV offers a more sensitive marker of cardiovascular risk in these populations and whether different QT correction methods yield consistent results. Longitudinal follow-up of this cohort will help determine whether repolarization parameters remain stable or change throughout childhood.

## Figures and Tables

**Table 1:** Baseline characteristics of participants by sex.

	Male	Female	*p*
*n*	60	79	
Age, years	12.5 ± 3.2	13.3 ± 3.5	0.159
**Waist, cm**	**90 ± 20**	**79 ± 19**	**<0.001**
**Systolic blood pressure, mmHg**	**122 ± 14**	**116 ± 13**	**0.015**
Diastolic blood pressure, mmHg	75 ± 9	74 ± 10	0.630
Triglycerides, mmol/L	0.9 (0.6–1.3)	0.9 (0.6–1.1)	0.698
HDL cholesterol, mmol/L	1.2 (1.0–1.5)	1.2 (1.0–1.4)	0.717
Fasting glucose, mmol/L	5.1 (4.7–5.2)	4.9 (4.6–5.2)	0.100
Sport activity, *n* (%)			0.091
30 min or less a day	16 (26.7%)	34 (43.0%)	
30–90 min a day	35 (58.3%)	39 (49.4%)	
90 min or more a day	9 (15.0%)	6 (7.6%)	
Social status, *n* (%)			0.203
Poor	3 (5.0%)	11 (13.9%)	
Average	47 (78.3%)	54 (68.4%)	
Good	10 (16.7%)	14 (17.7%)	

Data are given as the mean ± SD, median (IQR), or *n* (%). *p*-values were calculated using the Mann–Whitney U test, an independent-samples *t*-test, or the chi-square test as appropriate. HDL, high-density lipoprotein. Values in bold are considered to be statistically significant.

**Table 2:** Analyzed main effects by different ECG periods.

Parameter	F (df1, df2)	*p*-Value	η^2^ (Eta-Square)
RR			
**Age**	**54.06 (1131)**	**<0.001**	**0.292**
**Sex**	**9.87 (1131)**	**0.002**	**0.07**
**Metabolic syndrome**	**11.13 (1131)**	**0.001**	**0.078**
**Sport activity**	**4.92 (2131)**	**0.009**	**0.07**
Social status	0.13 (2131)	0.880	0.002
PR			
**Age**	**11.94 (1131)**	**<0.001**	**0.084**
Sex	2.14 (1131)	0.146	0.016
Metabolic syndrome	0.54 (1131)	0.463	0.004
Sport activity	0.05 (2131)	0.950	0.001
Social status	1.41 (2131)	0.247	0.021
QRS			
**Age**	**7.82 (1131)**	**0.006**	**0.056**
Sex	0.39 (1131)	0.533	0.003
Metabolic syndrome	1.83 (1131)	0.178	0.014
Sport activity	1.46 (2131)	0.237	0.022
Social status	0.56 (2131)	0.571	0.009
QTc			
**Age**	**10.15 (1131)**	**0.002**	**0.072**
**Sex**	**8.36 (1131)**	**0.004**	**0.06**
Metabolic syndrome	0.93 (1131)	0.336	0.007
Sport activity	0.33 (2131)	0.722	0.005
Social status	2.35 (2131)	0.100	0.035
Tte			
**Age**	**6.75 (1131)**	**0.010**	**0.049**
Sex	0.17 (1131)	0.685	0.001
Metabolic syndrome	3.9 (1131)	0.051	0.029
Sport activity	2.63 (2131)	0.076	0.039
Social status	0.93 (2131)	0.397	0.014
TP			
**Age**	**43.93 (1131)**	**<0.001**	**0.251**
**Sex**	**13.69 (1131)**	**<0.001**	**0.095**
**Metabolic syndrome**	**9.61 (1131)**	**0.002**	**0.068**
**Sport activity**	**3.71 (2131)**	**0.027**	**0.054**
Social status	0.39 (2131)	0.677	0.006

*p* < 0.05 was considered statistically significant. Effects in bold are statistically significant.

**Table 3:** Independent determinants of RR, QTc, and TP intervals.

Parameter	Mean Diff.	SE	*p*-Value	95% LCI	95% UCI
RR					
Sex *	64.708	20.597	0.002	23.962	105.453
Metabolic syndrome **	74.13	22.219	0.001	30.176	118.084
Sport activity					
30–90 min ***	48.96	22.413	0.031	4.263	93.298
>90 min ***	104.283	35.518	0.004	34.02	174.546
QTc					
Sex *	−10.227	3.537	0.004	−17.224	−3.23
TP					
Sex *	64.113	17.327	<0.001	29.836	98.39
Metabolic syndrome **	57.938	18.691	0.002	20.962	94.914
Sport activity					
30–90 min ***	38.955	18.854	0.041	1.657	76.254
>90 min ***	73.226	29.879	0.016	14.118	132.334

Estimated marginal means based on analysis of covariance model. *p* < 0.05 was considered statistically significant. SE, standard error; 95% LCI, 95% lower confidence interval; 95% UCI, 95% upper confidence interval. *: males compared with females; **: lack of metabolic syndrome compared with metabolic syndrome; ***: compared with less than 30 min/day of sport activity.

## Data Availability

The datasets used and/or analysed during the current study are available from the corresponding author on reasonable request.

## References

[1] Magnani J.W., Lopez F.L., Soliman E.Z., Maclehose R.F., Crow R.S., Alonso A. (2012). P Wave Indices, Obesity, and the Metabolic Syndrome: The Atherosclerosis Risk in Communities Study. Obesity.

[2] Altuncu M.E., Baspinar O., Keskin M. (2012). The use of short-term analysis of heart rate variability to assess autonomic function in obese children and its relationship with metabolic syndrome. Cardiol. J..

[3] Yagi R., Mori Y., Goto S., Iwami T., Inoue K. (2024). Routine Electrocardiogram Screening and Cardiovascular Disease Events in Adults. JAMA Intern. Med..

[4] Bagkaki A., Parthenakis F., Chlouverakis G., Galanakis E., Germanakis I. (2024). Cardiovascular Disease Screening in Primary School Children. Children.

[5] Vetter V.L. (2014). Electrocardiographic Screening of All Infants, Children, and Teenagers Should Be Performed. Circulation.

[6] Galobardes B., Smith G.D., Lynch J.W. (2006). Systematic Review of the Influence of Childhood Socioeconomic Circumstances on Risk for Cardiovascular Disease in Adulthood. Ann. Epidemiol..

[7] Dickinson D.F. (2005). The normal ECG in childhood and adolescence. Heart.

[8] World Health Organization (1995). Physical Status: The Use and Interpretation of Anthropometry, Report of a WHO Expert Committee.

[9] Zimmet P., Alberti K.G.M., Kaufman F., Tajima N., Silink M., Arslanian S., Wong G., Bennett P., Shaw J., Caprio S. (2007). The metabolic syndrome in children and adolescents? an IDF consensus report. Pediatr. Diabetes.

[10] Gupta P., Patel C., Patel H., Narayanaswamy S., Malhotra B., Green J.T., Yan G.-X. (2008). Tp-e/QT ratio as an index of arrhythmogenesis. J. Electrocardiol..

[11] Muensterman E.T., Tisdale J.E. (2018). Predictive Analytics for Identification of Patients at Risk for QT Interval Prolongation: A Systematic Review. Pharmacother. J. Hum. Pharmacol. Drug Ther..

[12] Zhang N., Gong M., Tse G., Zhang Z., Meng L., Yan B.P., Zhang L., Wu G., Xia Y., Xin-Yan G. (2018). Prolonged corrected QT interval in predicting atrial fibrillation: A systematic review and meta-analysis. Pacing Clin. Electrophysiol..

[13] Bhatia R.S., Bouck Z., Ivers N.M., Mecredy G., Singh J., Pendrith C., Ko D.T., Martin D., Wijeysundera H.C., Tu J.V. (2017). Electrocardiograms in Low-Risk Patients Undergoing an Annual Health Examination. JAMA Intern. Med..

[14] Force U.P.S.T., Curry S.J., Krist A.H., Owens D.K., Barry M.J., Caughey A.B., Davidson K.W., Doubeni C.A., Epling J.W., Kemper A.R. (2018). Screening for Cardiovascular Disease Risk With Electrocardiography. JAMA.

[15] Visseren F.L.J., Mach F., Smulders Y.M., Carballo D., Koskinas K.C., Bäck M., Benetos A., Biffi A., Boavida J.-M., Capodanno D. (2021). 2021 ESC Guidelines on cardiovascular disease prevention in clinical practice. Eur. Heart J..

[16] Corrado D., Basso C., Pavei A., Michieli P., Schiavon M., Thiene G. (2006). Trends in Sudden Cardiovascular Death in Young Competitive Athletes After Implementation of a Preparticipation Screening Program. JAMA.

[17] Zeppilli P., Biffi A., Cammarano M., Castelletti S., Cavarretta E., Cecchi F., Colivicchi F., Contursi M., Corrado D., D’aNdrea A. (2024). Italian Cardiological Guidelines (COCIS) for Competitive Sport Eligibility in athletes with heart disease: Update 2024. Minerva Medica.

[18] Kelly A.S., Barlow S.E., Rao G., Inge T.H., Hayman L.L., Steinberger J., Urbina E.M., Ewing L.J., Daniels S.R., American Heart Association Atherosclerosis (2013). Severe Obesity in Children and Adolescents: Identification, Associated Health Risks, and Treatment Approaches: A scientific statement from the American Heart Association. Circulation.

[19] Kaur A., Kaur N., Madhukar M. (2023). Assessment of Corrected QT Interval and QT Dispersion in Patients with Uncomplicated Metabolic Syndrome. J. Pharm. Bioallied Sci..

[20] Li W., Bai Y., Sun K., Xue H., Wang Y., Song X., Fan X., Song H., Han Y., Hui R. (2009). Patients with Metabolic Syndrome Have Prolonged Corrected QT Interval (QTc). Clin. Cardiol..

[21] Omran J., Bostick B.P., Chan A.K., Alpert M.A. (2018). Obesity and Ventricular Repolarization: A Comprehensive Review. Prog. Cardiovasc. Dis..

[22] Karaagac K., Tenekecioglu E., Yontar O.C., Kuzeytemiz M., Vatansever F., Tutuncu A., Ozluk O.A., Yilmaz M., Demir M. (2014). Effect of non-dipper and dipper blood pressure patterns on Tp-Te interval and Tp-Te/QT ratio in patients with metabolic syndrome. Int. J. Clin. Exp. Med..

[23] Rad E.M., Karimi M., Momtazmanesh S., Shabanian R., Saatchi M., Asbagh P.A., Zeinaloo A.A. (2021). Exercise-induced electrocardiographic changes after treadmill exercise testing in healthy children: A comprehensive study. Ann. Pediatr. Cardiol..

[24] Doumparatzi M., Sotiriou P., Deligiannis A., Kouidi E. (2023). Electrocardiographic characteristics of pediatric and adolescent football players. Sports Med. Health Sci..

[25] Llewellyn A., Simmonds M., Owen C.G., Woolacott N. (2015). Childhood obesity as a predictor of morbidity in adulthood: A systematic review and meta-analysis. Obes. Rev..

[26] Owen C.G., Whincup P.H., Orfei L., Chou Q.-A., Rudnicka A.R., Wathern A.K., Kaye S.J., Eriksson J.G., Osmond C., Cook D.G. (2009). Is body mass index before middle age related to coronary heart disease risk in later life? Evidence from observational studies. Int. J. Obes..

[27] A El Sehmawy A., Fawaz R.A.E.S., Agiba N.A., Elsherbiny E.A., Agaba N.F., Mohammed D.S., Nasr H.M., Diab F.E.A., Ahmed A.M., Mahfouz S.I. (2025). Impact of Different Metabolic Indicators on Ventricular Repolarization Indices in Obese Children: A Case Control Study. Clin. Med. Insights Endocrinol. Diabetes.

[28] Kiess A., Körner A., Dähnert I., Vogel M., Markel F., Gebauer R.A., Kiess W., Paech C. (2020). Does obesity have an effect on the ECG in children?. J. Pediatr. Endocrinol. Metab..

[29] Cordeiro J.R., Mosca S., Correia-Costa A., Ferreira C., Pimenta J., Correia-Costa L., Barros H., Postolache O. (2023). The Association between Childhood Obesity and Cardiovascular Changes in 10 Years Using Special Data Science Analysis. Children.

[30] Luca A.C., Țarcă E., Tănase V.-G., Pădureț I.-A., Dragoiu T.-S., Butnariu L.I., Roșu S.T., Roca I.C., Mîndru D.-E. (2024). Benefits of Physical Activity in Children with Cardiac Diseases—A Concise Summary for Pediatricians. Children.

[31] McClean G., Riding N.R., Ardern C.L., Farooq A., E Pieles G., Watt V., Adamuz C., George K.P., Oxborough D., Wilson M.G. (2017). Electrical and structural adaptations of the paediatric athlete’s heart: A systematic review with meta-analysis. Br. J. Sports Med..

[32] Leppänen M.H., Haapala E.A., Veijalainen A., Seppälä S., Oliveira R.S., Lintu N., Laitinen T., Tarvainen M.P., Lakka T.A. (2019). Associations of cardiometabolic risk factors with heart rate variability in 6- to 8-year-old children: The PANIC Study. Pediatr. Diabetes.

[33] E Speer K., Naumovski N., McKune A.J. (2024). Heart rate variability to track autonomic nervous system health in young children: Effects of physical activity and cardiometabolic risk factors. Physiol. Behav..

[34] Chen H., Xu J., Xie H., Huang Y., Shen X., Xu F. (2022). Effects of physical activity on heart rate variability in children and adolescents: A systematic review and meta-analysis. Cienc. Saude Coletiva.

[35] Stringhini S., Dugravot A., Kivimaki M., Shipley M., Zins M., Goldberg M., Ferrie J.E., Singh-Manoux A. (2010). Do different measures of early life socioeconomic circumstances predict adult mortality? Evidence from the British Whitehall II and French GAZEL studies. J. Epidemiol. Community Health.

[36] Hulmán A., Tabák A.G., A Nyári T., Vistisen D., Kivimäki M., Brunner E.J., Witte D.R. (2014). Effect of secular trends on age-related trajectories of cardiovascular risk factors: The Whitehall II longitudinal study 1985–2009. Leuk. Res..

[37] Bijker R., Agyemang C. (2015). The influence of early-life conditions on cardiovascular disease later in life among ethnic minority populations: A systematic review. Intern. Emerg. Med..

[38] Boylan J.M., Jennings J.R., Matthews K.A. (2016). Childhood socioeconomic status and cardiovascular reactivity and recovery among Black and White men: Mitigating effects of psychological resources. Heal. Psychol..

[39] Benatar A., Decraene T. (2001). Comparison of formulae for heart rate correction of QT interval in exercise ECGs from healthy children. Heart.

